# Beyond the Stroke: A Case Report of Multiple Sclerosis

**DOI:** 10.7759/cureus.73962

**Published:** 2024-11-18

**Authors:** Anjali N Karri, Attika Khalid, Karthik Vasudevan Iyer

**Affiliations:** 1 Department of Acute Medicine, Good Hope Hospital, Birmingham, GBR; 2 Department of Anaesthetics and Intensive Treatment Unit (ITU), Birmingham Heartlands Hospital, Birmingham, GBR

**Keywords:** and paresthesia, anti-cd20 monoclonal antibodies, mri brain and spine, multiple sclerosis and other demyelinating disorders, multiple sclerosis behavior, radiological findings in demyelination, stroke, systemic steroids, weakness in limbs

## Abstract

Multiple sclerosis is an inflammatory, autoimmune demyelinating condition and poses diagnostic challenges due to varied presentations. This case report presents a divergence from typical clinical presentations of multiple sclerosis (MS), as the initial presentation resembled symptoms of a brain stem stroke. Conventionally, MS suspicion arises in the presence of previous neurological deficits or signs of optic neuritis. This case emphasises the need for high suspicion of MS in a suspected stroke or transient ischaemic attack (TIA).

A 29-year-old woman presented with symptoms characterised by crossed hemiparesis, reduced coarse touch sensation, and paraesthesia of face and upper limb, which initially mimicked symptoms of a brain stem stroke with a normal CT head. MRI revealed multiple hyperintense lesions indicative of demyelinating disease, likely MS. MRI contrast did not identify active lesions. Further investigations included a vasculitis screen, MOG antibodies, and serum angiotensin-converting enzyme (ACE) levels. Treatment with intravenous methylprednisolone for working diagnosis of clinically isolated syndrome (CIS), a classification subset of MS, resulted in symptomatic improvement. The patient responded well and had a lumbar puncture in neurology follow-up, confirming relapsing and remitting multiple sclerosis (RRMS) and was started on disease-modifying treatment (DMT).

Diagnosis of MS relies on McDonald criteria, emphasising dissemination in time and space. Early intervention and familiarity with diagnostic criteria are crucial when dealing with MS. This case highlights the diagnostic complexities of MS and the importance of a comprehensive approach to clinical evaluation. A young patient presenting with progressive symptoms affecting activities of daily living should prompt urgent investigations for early initiation of treatment. Familiarity with diagnostic criteria and treatment options optimises patient care in MS management.

## Introduction

Multiple sclerosis (MS) is conventionally suspected in patients in the presence of previous episodes of neurological deficits or signs of optic neuritis, which occur in approximately 15%-20% of initial presentations [1]. Multiple sclerosis as the first presentation can depict sensory disturbances and motor symptoms mimicking a stroke or TIA and leading to misdiagnosis and treatment [2].

An estimated 2.8 million individuals worldwide are affected by MS, with an incidence of at least twice in females than in males, a ratio ranging from 2:1 to 4:1 depending on geography, with a mean age of 32 years at the time of diagnosis [3]. Acute MS lesions can present as pseudo-strokes, leading to misdiagnosis [4,5]. This case presents a 29-year-old female with atypical clinical presentations of MS characterised by crossed hemiparesis and reduced coarse touch sensation with hyperalgesia, which mimicked symptoms of a brain stem stroke. This intends to emphasise the possibility of diverse clinical presentations and the rationale to consider broader differentials.

## Case presentation

A 29-year-old female patient, previously fit and well, initially presented to the emergency department with headache, weakness, and altered sensations on the left side of the body and reduced sensation on the right side of the face for one week. Past medical history of inflammatory bowel disease and family history were insignificant for neurological pathologies; she has a desk job and no history of smoking or alcohol consumption. A CT head was done for suspected stroke or space-occupying lesion (SOL), which was unremarkable, and the patient was self-discharged.

She was referred four days later by her general physician (GP) to our hospital medical assessment unit (MAU) with right-sided headache, left-sided tingling, and worsening weakness. Initial blood workup showed all parameters within the normal range except for an erythrocyte sedimentation rate (ESR) of 118. Clinical examination revealed reduced touch sensation on the right side of the face (maxillary branch territory of the trigeminal nerve). Diminished touch sensation and reduced power in the Medical Research Council Scale for muscle strength (MRC) of 4/5 in the left upper and lower limb. Bilateral deep tendon reflexes were present. There were no speech abnormalities, visual field defects, painful eye movements, altered color vision or nystagmus. Fundoscopy was normal bilaterally. There was no dysdiadochokinesia. Romberg sign was negative. A repeat CT head ruled out any acute intracranial pathology and was started on high-dose aspirin as clinical ischemic stroke (pontine) with differentials of multiple sclerosis and vasculitis. Blood sample was sent for a vasculitis screen, and the patient was discharged to follow up with an MRI as an outpatient.

She returned to the hospital two days later with progressive weakness affecting her work and was admitted to the acute medicine department for in-patient MRI.

**Figure 1:**
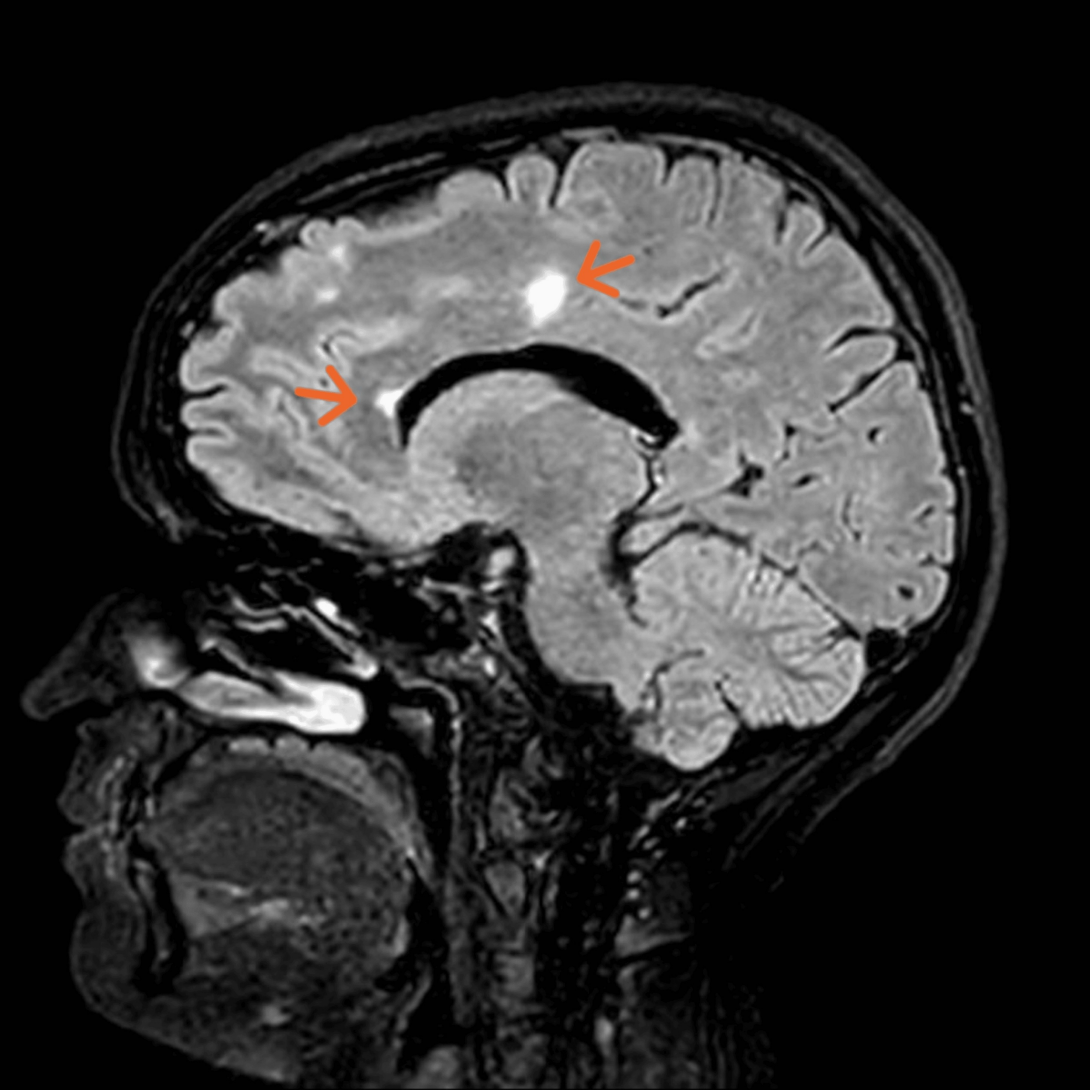
Sagittal midline MRI FLAIR image of the head showing oval-shaped periventricular and peri-callosal variable sized lesions. MRI: magnetic resonance imaging FLAIR: fluid-attenuated inversion recovery

**Figure 2:**
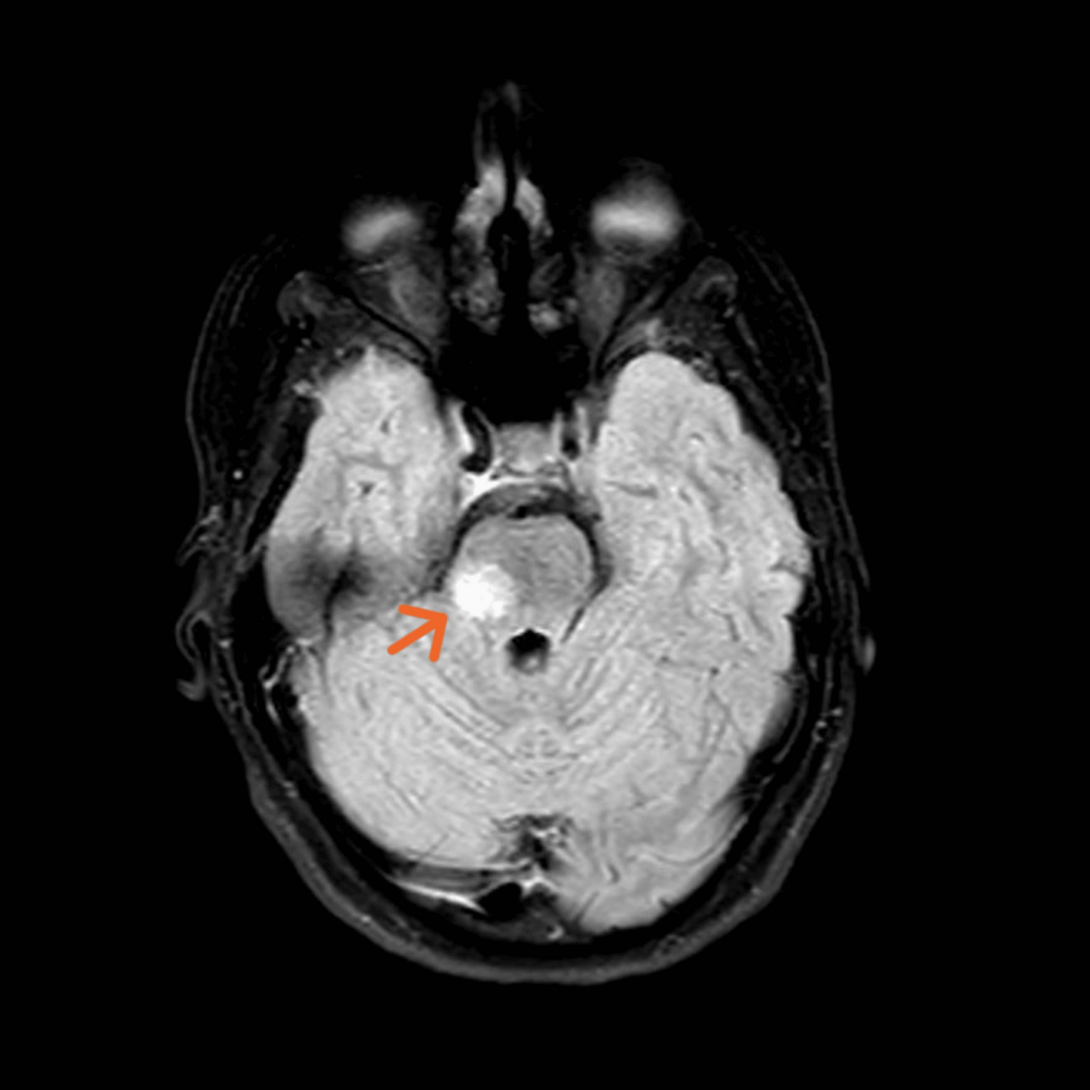
Axial FLAIR MRI of the head at the level of pons showing lesion on the right side of the pons and right middle cerebellar peduncle. FLAIR: fluid-attenuated inversion recovery

MRI revealed multiple oval-shaped periventricular and peri-callosal lesions of high T2 signal intensity bilaterally, with a lesion on the right side of the pons and right middle cerebellar peduncle with no restriction (Figures 1, 2), significant for demyelinating disease, likely MS. The patient was reluctant to proceed with lumbar puncture (LP) (for evidence of oligoclonal bands) [6], and it was decided to perform an MRI with contrast to identify active lesions to demonstrate dissemination in time as per the MS diagnostic criteria (McDonald criteria) [7]. However, no such lesions were found. The MRI of the whole spine with contrast was unremarkable as well.

Post-MRI, the neurologist was consulted and decided to treat it as a clinically isolated syndrome (CIS) and was started on 1 g/day IV (intravenous) methylprednisolone for three days to manage the acute symptoms (possible flare).

Due to atypical presentation, further investigations for differential diagnosis, a vasculitis screen, myelin oligodendrocyte glycoprotein (MOG) antibodies and serum angiotensin-converting enzyme (ACE) level investigations were done.

The patient responded well to the treatment; pain and touch sensation were significantly improved with a slight improvement in power. Neuro-physiotherapy was done during the inpatient stay and was discharged with an outpatient (OP) neurology clinic appointment for consideration of lumbar puncture and disease-modifying treatment (DMT).

Two weeks later, she was reviewed in clinic with the recurrence of left foot and hand numbness, and new bladder urgency and frequency; amitriptyline was commenced. The patient then consented for LP, and cerebrospinal fluid (CSF) showed the presence of oligoclonal bands with none in serum. Other CSF tests were normal (white blood count (WBC) < 1, no organism on staining, no growth).

The lab results of complement, dsDna, and serum ACE were within normal limits, anti-MOG antibodies negative, and aquaporin 4 antibody negative. MPO abs and PR3 abs are negative with normal vitamin B12 and folate levels.

A multidisciplinary team meeting (MDT) was held, and a diagnosis of relapsing, remitting multiple sclerosis (RRMS) was confirmed with EDSS (Expanded Disability Status Scale) score of 0 (Table 1). And she was started on treatment with disease-modifying drugs Ocrevus and Kesimpta. The patient followed up with MS nurses, regular physiotherapy and planned for a review appointment with the neurologist.

**Table 1:** EDSS (Expanded Disability Status Scale) scoring *Permission was obtained to replicate the table from* the *Multiple Sclerosis Trust website [8].* MS: Multiple sclerosis

Score	Disability
0	No disability in functional status
1.0-3.5	Minimal–moderate disability
4.0-6.5	Significant disability-dependent on walking aids
7.0-8.0	Wheelchair use – restricted to bed
9.0-9.5	Confined to bed
10.0	Death due to MS

## Discussion

Multiple sclerosis is an inflammatory, autoimmune demyelinating condition of the central nervous system that affects the myelin sheath, causing sclerosis [9]. It is diagnosed based on McDonald's criteria, which emphasises the presence of “dissemination in space" (DIS) and “dissemination in time" (DIT) (Table 2).

In our case, the absence of stroke risk factors, along with the determinants of young age and female sex, led to the suspicion of an autoimmune illness such as MS.

**Table 2:** 2017 revised McDonald criteria: evidence needed to secure an MS diagnosis Permission was obtained to replicate the table from the Multiple Sclerosis Trust website [10]. MS: Multiple sclerosis; DIT: dissemination in time; DIS: dissemination in space; CIS: clinically isolated syndrome; PPMS: primary progressive multiple sclerosis In addition to the above, the 2024 revised criteria would classify patients as radiologically isolated syndrome (RIS) [11].

Existing evidence of patient	Additional evidence needed to establish the diagnosis of MS
Two or more relapses AND EITHER objective clinical evidence of two or more lesions OR of one lesion with previous relapse.	None
Two or more relapses; objective clinical evidence of one lesion (establishing DIT)	DIS : One or more MRI-detected lesions typical of MS OR A further relapse showing damage to another part of the CNS.
One relapse; objective clinical evidence of two or more lesions (establishing DIS)	DIT: Oligoclonal bands OR New lesion on MRI in addition to existing lesions OR A further relapse.
One attack; objective clinical evidence of one lesion (establishing CIS)	DIS: One or more MRI-detected lesions typical of MS OR A further relapse of activity in another part of the CNS. DIT: Oligoclonal bands OR new lesions in MRI OR A further relapse.
Progression of neurological symptoms of MS (PPMS)	One year of progression plus any two of: One or more MRI lesions in the brain typical of MS, Two or more MRI lesions in the spinal cord, oligoclonal bands in the spinal fluid.

**Figure 3:**
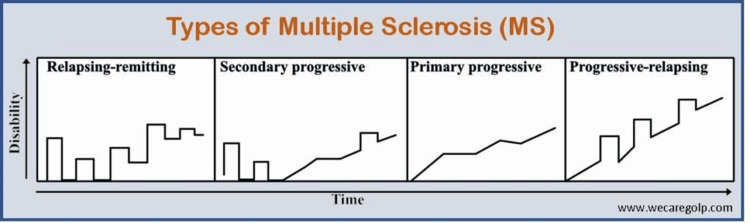
Types of multiple sclerosis: pattern of clinical presentation Permission was obtained to replicate the figure from We Care: The Health Academy [13].

Based on clinical course, MS is classified into relapsing, remitting type (RRMS) (the most common type), primary progressive (PPMS), secondary progressive (SPMS), and progressive relapsing (PRMS) types (Figure 3). With evidence of a significant proportion of RRMS, patients over the course progress into SPMS [12].

Clinically isolated syndrome (CIS) is an episode of neurologic symptoms that are the first clinical sign of possible MS. Often, CIS is misdiagnosed for other neurological conditions due to its insidious onset and lack of a prior history of neurological deficits. In our patient, the dissemination in space was evident by MRI findings, and to evaluate the dissemination in time criteria, an MRI contrast was done. As the patient had started IV methylprednisolone before the MRI contrast (reduced sensitivity of contrast uptake), the diagnosis of MS could not yet be established. Hence, CIS was the initial working diagnosis. Further evidence to meet McDonald criteria was gathered by lumbar puncture and flare of symptoms to confirm RRMS.

MRI findings of hyperintense lesions are not exclusive for multiple sclerosis, as other demyelinating disorders have similar imaging results [7]. Further investigations are required to rule out differentials of the MRI findings of multiple sclerosis (Table 3).

**Table 3:** Differentials of MRI findings of multiple sclerosis CNS: Central nervous system; ESR: erythrocyte sedimentation rate; CRP: C-reactive protein; ANCAs: anti-neutrophilic cytoplasmic antibodies; MOG: myelin oligodendrocyte; ACE: angiotensin-converting enzyme; AQP4-IgG: aquaporin-4 immunoglobulin G antibodies [14-16] Original table compilation.

S.No	Differential Diagnosis	Investigation
1.	CNS vasculitis	Vasculitis screen (ESR, CRP, ANCAs, rheumatoid factor, complement protein, coagulation factors)
2.	Myelin Oligodendrocyte Glycoprotein Antibody-associated Disease (MOGAD)	MOG antibodies
3.	Neuro-sarcoidosis	Serum ACE levels, tissue biopsy
4.	Neuromyelitis optica (NMO)	AQP4-IgG antibodies

In our case, the differentials have been ruled out by extensive workup, and the diagnosis of multiple sclerosis was established. The delay in diagnosis was due to discharge with planned follow-up instead of inpatient MRI and patient reluctance for LP.

Currently, there is no cure for MS, and the main goal of treatment is to manage flare-ups, reduce relapses and delay disease progression. Treatment is based on the EDSS score with management of acute flare by corticosteroids and reduction of frequency of attacks by DMT (disease-modifying treatment). Flare-up is defined as a relapse of MS if there are new symptoms or worsening of existing symptoms lasting for more than 24 hours in the absence of any other cause after a period of one month [17]. High-dose steroids and/or plasmapheresis are the mainstay of treatment for flare-ups [18].

Multiple Food and Drug Administration (FDA)-approved medications are available as disease-modifying treatment (DMT), including interferon beta, galtiramer acetate, sphingosine-1-phosphate (S1P) receptor modulators, integrin inhibitors and monoclonal antibodies [19].

Monoclonal antibodies (mAbs) were developed to target the CD-20 antigen of B cells, of which Ocrevus (Ocrelizumab) and Kesimpta (Ofatumumab) opted for our patient are humanised mAbs, given as intravenous and subcutaneous injections respectively, and known to a have good safety index [20].

## Conclusions

This case emphasised the diagnostic complexity of multiple sclerosis (MS), particularly in patients presenting with atypical symptoms. Our patient presented with neurological symptoms suggestive of stroke, and after extensive evaluation, including MRI and lumbar puncture, the diagnosis of relapsing-remitting multiple sclerosis (RRMS) was confirmed. Identification of oligoclonal bands has been a key diagnostic feature in this case. Currently there is no definitive cure for MS; early diagnosis and treatment improve long-term outcomes for patients.
